# Measurable residual mutated *NPM1* before allogeneic transplant for acute myeloid leukemia

**DOI:** 10.1038/s41409-025-02757-1

**Published:** 2025-11-24

**Authors:** Rasha W. Al-Ali, Gege Gui, Niveditha Ravindra, Georgia Andrew, Devdeep Mukherjee, Zoë C. Wong, Ying Huang, Jason Gerhold, Matt Holman, Austin Jacobsen, Julian D’Angelo, Jeffrey Miller, Karina Elias, Jeffery J. Auletta, Firas El Chaer, Steven M. Devine, Antonio Martin Jimenez Jimenez, Marcos J. G. De Lima, Mark R. Litzow, Partow Kebriaei, Wael Saber, Stephen R. Spellman, Scott L. Zeger, Kristin M. Page, Jerald P. Radich, R. Coleman Lindsley, Laura W. Dillon, Christopher S. Hourigan

**Affiliations:** 1 Fralin Biomedical Research Institute, Virginia Tech FBRI Cancer Research Center, Washington, DC USA; 2 Laboratory of Myeloid Malignancies, Hematology Branch, National Heart, Lung, and Blood Institute, National Institutes of Health, Bethesda, MD USA; 3 Invivoscribe, Inc, San Diego, CA USA; 4 Center for International Blood and Marrow Transplant Research, NMDP, Minneapolis, MN USA; 5 The Ohio State University College of Medicine, Columbus, OH USA; 6 University of Virginia, Charlottesville, VA USA; 7 Sylvester Comprehensive Cancer Center, Miami, FL USA; 8 Mayo Clinic, Rochester, MN USA; 9 The University of Texas MD Anderson Cancer Center, Houston, TX USA; 10 Center for International Blood and Marrow Transplant Research, Medical College of Wisconsin, Milwaukee, WI USA; 11 Department of Biostatistics, Johns Hopkins Bloomberg School of Public Health, Baltimore, MD USA; 12 Fred Hutchinson Cancer Research Center, Seattle, WA USA; 13 Dana-Farber Cancer Institute, Harvard Medical School, Boston, MA USA

**Keywords:** Translational research, Acute myeloid leukaemia, Stem-cell therapies

*NPM1* mutations (*NPM1*m), seen in ~30% of adults with acute myeloid leukemia (AML), often co-occur with *FLT3* internal tandem duplication (*FLT3*-ITD) [1, 2]. Measurable residual disease (MRD) testing in first complete remission (CR) identifies patients at high risk of relapse after allogeneic hematopoietic cell transplantation (alloHCT) [3, 4]. Quantitative RT-PCR assays are currently recommended for *NPM1* MRD testing, while *FLT3*-ITD MRD testing is DNA-based using next generation sequencing (NGS) [5–7]. DNA-based NGS assays for *NPM1* MRD testing may offer advantages over RNA, including improved template stability, accurate quantification, and high throughput capabilities, but require validation. We performed *NPM1* MRD detection in blood collected from patients with AML during first CR (CR1) prior to alloHCT using a highly sensitive, commercially available DNA-based *NPM1* NGS MRD assay.

A randomly selected subset of 190 patients aged 18 or older from the Pre-MEASURE study [3] who received an alloHCT for *NPM1* mutated AML during CR1 in the USA between 2013-2019 were included in this analysis (Supplementary Table 1; Supplementary Figs. 1 and 2). Patients provided written informed consent for participation in the NMDP institutional review board-approved CIBMTR database (NCT01166009) and biorepository (NCT04920474) protocols. Research was performed in compliance with all applicable federal regulations pertaining to the protection of human research participants and with approval of the CIBMTR observational research group.

A commercially available research testing kit (IVS, Invivoscribe, San Diego, CA), which can detect DNA-based *NPM1* MRD, was used to establish a workflow following clinical testing standards and validated to detect *NPM1* insertion variants down to a variant allele fraction (VAF) of at least 0.005% (Supplementary Fig. 3). Results were compared to those previously reported using an anchored multiplex PCR-based (AMP) targeted NGS assay (detection limit 0.01%) and, where applicable, *FLT3*-ITD MRD results. Additional details are provided in the Supplementary Methods.

*NPM1* NGS MRD testing was successful for 186 of 190 eligible (98%) patients, of which 48 (26%) relapsed (median: 4.0 months; range: 0.7-52.5) and 64 (34%) died (median: 9.7 months; range: 0.4-58.9) post-alloHCT. The IVS assay detected *NPM1* insertion variants in 71 (38%) patients with a median VAF of 0.0026% (range:0.0002-2.1%) (Supplementary Table 2).

Using the previously reported VAF threshold of 0.01%, the concordance between the IVS and the AMP assays was 96% (*n* = 21 as both positive and *n* = 158 as both negative) (Supplementary Fig. 4). Using this threshold, 24 patients tested positive for *NPM1* MRD pre-alloHCT by the IVS test, which was associated with significantly increased rates of relapse (52% vs. 20% at 3 yrs; HR = 4.3; *P* < 0.001) and decreased OS (34% vs. 71% at 3 yrs; HR = 3.6; *P* < 0.001) compared to those testing negative, in close alignment with the clinical outcomes previously reported using the AMP assay (Supplementary Fig. 5).

The IVS test, however, has an increased sensitivity compared with the previously reported AMP test, allowing confident detection of *NPM1*m below a VAF of 0.01%. Removing this threshold (ie: any *NPM1*m detection considered to be MRD positivity) dropped the concordance between the IVS and the AMP assays to 76% (109 cases reported as both negative and 33 as both positive) and resulted in additional patients being classified as *NPM1* MRD positive by the IVS test. Patients identified as *NPM1*m positive in CR1 blood prior to alloHCT by the IVS test (n = 71, 38%) had increased rates of relapse (40% vs. 15% at 3 yrs; HR = 3.4; *P* < 0.001) and decreased OS (50% vs. 75% at 3 yrs; HR = 2.9; *P* < 0.001) after transplant compared with those testing negative (Supplementary Fig. 5). When considering additional VAF thresholds, patients in the highest VAF group (≥0.1%) had the worst clinical outcomes compared to patients in other groups (relapse 60%, OS 27%, at 3 yrs) (Fig. 1a). In multivariable analysis, *NPM1*m MRD burden in CR1 blood prior to alloHCT was also associated with increased risk of relapse and death in a dose-dependent manner, with a VAF of 0.01% or greater associated with the highest risk (Supplementary Fig. 6).

**Fig. 1:**
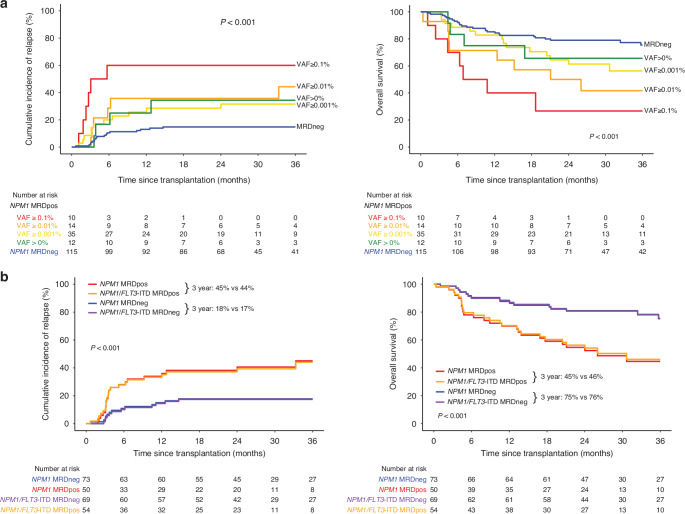
Association of *NPM1* and/or *FLT3*-ITD measurable residual disease (MRD) and clinical outcomes in patients with acute myeloid leukemia (AML) prior to allogeneic hematopoietic cell transplant. Cumulative incidence of relapse (left) and overall survival (OS, right) are shown for patients based on the presence (MRDpos) or absence (MRDneg) of *NPM1* and/or *FLT3-*ITD variants detected by the Invivoscribe MRD next generation sequencing assays. **a** All patients with *NPM1*-mutated AML grouped by *NPM1* MRD thresholds. **b** Patients co-mutated for *NPM1* and *FLT3*-ITD at baseline grouped by the *NPM1* MRD status versus *NPM1* and/or *FLT3*-ITD MRD status.

Patients were further divided based on their reported *FLT3*-ITD mutational status at baseline. A total of 123 patients (66%) were co-mutated for *FLT3*-ITD at baseline. Increased risk of relapse was seen for *NPM1*m MRD positive (*NPM1*m VAF > 0) patients regardless of their baseline *FLT3*-ITD status. For those without *FLT3*-ITD at baseline, patients testing MRD positive for *NPM1* by IVS had increased CIR (29% vs. 10% at 3 yrs; HR = 4.4; *P* = 0.01) compared to those testing negative; for those with *FLT3*-ITD at baseline, the same trend was observed (45% vs. 18% at 3 yrs; HR = 2.8; *P* = 0.003) (Supplementary Fig. 7).

For patients with mutations in both *NPM1* and *FLT3*-ITD reported at baseline, *FLT3*-ITD MRD was previously performed using the IVS assay [4]. Defining MRD based on the presence of *NPM1* vs. *NPM1* and/or *FLT3*-ITD revealed equivalent CIR (45% vs. 44%) and OS (45% vs. 46%) at 3 years. 54 were MRD positive for *NPM1* and/or *FLT3*-ITD (22 *NPM1* MRD positive only, and 4 *FLT3*-ITD MRD positive only). (Fig. 1b). The MRD negative groups also had similar CIR (18% vs. 17%) and OS (75% vs. 76%) at 3 years, and the MRD positive group by either definition had inferior clinical outcomes (CIR and OS) compared to those in the corresponding MRD negative group (*P* < 0.001 for both outcomes under two definitions). We next evaluated the prognostic value of each test alone. A total of 6 relapse events were identified only by *NPM1* MRD testing and 1 relapse event only by *FLT3*-ITD MRD testing; with equivalent CIR at 3 years for the two tests (45% vs. 64%, *NPM1* vs. *FLT3*-ITD, *P* = 0.237), (Supplementary Fig. 8). These results were confirmed in the full Pre-MEASURE cohort for co-mutated patients (*n* = 317) using the AMP assay, where 13 and 1 additional relapse events were identified using *NPM1* and *FLT3*-ITD MRD testing, respectively, and equivalent CIR at 3 years (70% vs. 73%, *NPM1* vs. *FLT3*-ITD, *P* = 0.499).

In this study evaluating patients with *NPM1* mutated AML from the Pre-MEASURE cohort, we show that the detection of *NPM1*m in pre-transplant blood during first complete remission using a highly sensitive DNA-based assay is associated, in a dose-dependent manner, with a significantly increased risk of relapse and death after alloHCT. Some of this risk could be mitigated by conditioning regimen, with higher intensity regimens being associated with decreased relapse and improved survival in *NPM1* MRD positive patients (Supplementary Fig. 9).

Similar to *FLT3*-ITD [4], an *NPM1* MRD VAF threshold of ≥0.01% pre-transplant identified patients at greatest risk of relapse and death. Current guidelines for *NPM1* MRD testing have been based on measuring mutant RNA transcript levels [5], additional analytical and clinical utility validation work is needed to compare DNA and RNA based *NPM1*m MRD testing thresholds [8, 9].

*NPM1* and *FLT3*-ITD mutations often co-occur, presenting multiple potential targets for MRD tracking. In patients co-mutated for both *FLT3*-ITD and *NPM1* at diagnosis, we found that tracking *NPM1m* showed equivalent ability to identify relapsing patients comparing to tracking mutations in both *NPM1* and *FLT3*-ITD or *FLT3*-ITD alone pre-alloHCT. Our observation that very few co-mutated patients test MRD positive for *FLT3*-ITD but not *NPM1*m is supported by data from other studies [9–11]. Therefore, while there may be additional important predictive use cases for *FLT3*-ITD NGS-MRD testing, our results support findings that validate the prognostic value of pre-transplant *NPM1* MRD and support prioritizing it over *FLT3-ITD* when only one test is available [12].

## Supplementary information

Supplemental Material

## Data Availability

FASTQ files are available in the NCBI Sequence Read Archive (SRA) (accession: PRJNA1140149).
