# Supplementary material for: Measurable residual mutated NPM1 before allogeneic transplant for acute myeloid leukemia
Source: Bone Marrow Transplant. 2025 Nov 24;61(2):222–4. doi: 10.1038/s41409-025-02757-1 (PMC12909118; doi:10.1038/s41409-025-02757-1)
Supplement: Supplementary file 1 — Supplemental Material [file 41409_2025_2757_MOESM1_ESM.pdf]

Supplementary Data

Measurable Residual Mutated *NPM1* before Allogeneic Transplant for Acute Myeloid Leukemia

Rasha W Al-Ali[1]*, Gege Gui[1]*, Niveditha Ravindra[2], Georgia Andrew[2], Devdeep Mukherjee[2], Zoë C Wong[2], Ying Huang[3], Jason Gerhold[3], Matt Holman[3], Austin Jacobsen[3], Julian D'Angelo[3], Jeffrey Miller[3], Karina Elias[3], Jeffery J Auletta[4,5], Firas El Chaer[6], Steven M Devine[4], Antonio Martin Jimenez Jimenez[7], Marcos J G De Lima[5], Mark R Litzow[8], Partow Kebriaei[9], Wael Saber[10], Stephen R Spellman[4], Scott L Zeger[11], Kristin M Page[10], Jerald P Radich[12], R Coleman Lindsley[13], Laura W Dillon[1#], Christopher S Hourigan[1#]

[1]Fralin Biomedical Research Institute, Virginia Tech FBRI Cancer Research Center, Washington, DC
[2]Laboratory of Myeloid Malignancies, Hematology Branch, National Heart, Lung, and Blood Institute, National Institutes of Health, Bethesda, MD
[3]Invivoscribe, Inc., San Diego, CA
[4]Center for International Blood and Marrow Transplant Research, NMDP, Minneapolis, MN
[5]The Ohio State University College of Medicine, Columbus, OH
[6]University of Virginia, Charlottesville, VA
[7]Sylvester Comprehensive Cancer Center, Miami, FL
[8]Mayo Clinic, Rochester, MN
[9]The University of Texas MD Anderson Cancer Center, Houston, TX
[10]Center for International Blood and Marrow Transplant Research, Medical College of Wisconsin, Milwaukee, WI
[11]Department of Biostatistics, Johns Hopkins Bloomberg School of Public Health, Baltimore, MD
[12]Fred Hutchinson Cancer Research Center, Seattle, WA
[13]Dana-Farber Cancer Institute, Harvard Medical School, Boston, MA

Table of Contents

Supplementary Methods	Page 2
Supplementary Figure 1. Patient selection flowchart	Page 4
Supplementary Figure 2. Sample size selection	Page 5
Supplementary Figure 3. Validation of the Invivoscribe *NPM1* MRD assay	Page 6
Supplementary Figure 4. VAF comparison between IVS and AMP assays	Page 7
Supplementary Figure 5. *NPM1* MRD status for *NPM1* mutated patients and the association with clinical outcomes after allogeneic hematopoietic cell transplant	Page 8
Supplementary Figure 6. Multivariable regression analyses.	Page 9
Supplementary Figure 7. *NPM1* MRD status for *NPM1* mutated patients by IVS using a VAF threshold of >0% and the association with clinical outcomes after allogeneic hematopoietic cell transplant considering baseline *FLT3*-ITD mutations	Page 10
Supplementary Figure 8. NGS MRD status for *NPM1* and *FLT3*-ITD mutated patients and the association with clinical outcomes after allogeneic hematopoietic cell transplant	Page 11
Supplementary Figure 9. NGS MRD status for all *NPM1* mutated patients from the PreMEASURE study and the association with clinical outcomes after allogeneic hematopoietic cell transplant stratified by conditioning intensity	Page 12
Supplementary Table 1. *NPM1*-mutated AML patient baseline clinical characteristics	Page 13
Supplementary Table 2. Residual *NPM1* and *FLT3*-ITD Variants Detected in the Blood of AML Patients Prior to Transplant.	Page 14
Supplementary References	Page 15

Supplementary Methods

Samples and DNA Isolation

Pre-transplant, pre-conditioning baseline whole blood samples from *NPM1* mutated AML patients from the Pre-MEASURE study were collected within 100 days prior to transplant and high-quality genomic DNA (gDNA) extracted as previously described.[1] Patients were eligible for re-analysis in this study if they had at least 700ng of gDNA remaining and DNA concentration ≥20ng/μL. Patients were randomly selected from those eligible as described in Supplementary Figure 2.

NPM1 targeted next-generation sequencing (NGS)

NPM1 exon 12 insertion variants were detected using the Invivoscribe *NPM1* MRD assay (Invivoscribe Inc., San Diego, CA) following manufacturer's instructions. In short, 700ng of gDNA was amplified by polymerase chain reaction on a Mastercycler X50a (Eppendorf AG, Hamburg, Germany) using oligonucleotides designed with Illumina adapters containing unique molecular indices targeting the exon 12 region of the *NPM1* gene. Following PCR amplification, the PCR products were subjected to two rounds of cleanup using Ampure XP reagent (Beckman Coulter Inc., Brea, CA) on a Zephyr G3 NGS Workstation (PerkinElmer Health Sciences, Inc., Shelton, CT). Samples were processed in batches of 24, including 21 patient samples, a positive control, a negative control, and a no-template control (NTC). Resulting libraries were subjected to quality control evaluation using the D1000 screentape assay on the Tapestation 4200 instrument (Agilent Technologies). A run passed if patient samples and positive/negative controls had peaks between 200-1000bp with a concentration >1.0 ng/μL and <1.0ng/μL for NTC.

Equal molar quantities of each library were pooled and subjected to paired-end 300-bp sequencing on the MiSeq instrument (Ilumina). A sequencing run passed if the MiSeq™ Cluster Density was ≥500 K/mm^2, the total MiSeq™ Run Q30 Score was ≥60%, and at least 10 million MiSeq™ reads passed filter.

Data analysis was performed on demultiplexed FASTQ files using the *NPM1* MRD v1.1.1 Software (Invivoscribe, Inc.) using the NIH HPC Biowulf cluster (http://hpc.nih.gov). If the positive control, negative

control, or NTC samples did not pass, the protocol was repeated from the beginning for all samples. If any sample was not evaluable, the protocol was repeated from the beginning for that sample.

For assay validation, 21 competency standards were created by Invivoscribe, Inc. consisting of serial dilutions of *NPM1* cell line and patient sample positive controls for types A, B, D, and other and targeted variant allele fractions (VAFs) ranging from 1% to 0.0001%. Competency standards were analyzed in singlet at both the National Institutes of Health laboratory and the Invivoscribe, Inc. laboratory. All samples above the validated LOD of 0.005% VAF were confirmed by both laboratories, with highly consistent results (Supplementary Figure 3).

Statistical Methods

Overall survival (OS) and cumulative incidence of relapse (CIR) with non-relapse mortality (NRM) as competing risk were estimated, considering the day of transplant as time 0 by Kaplan-Meier estimation (log-rank tests), Fine and Gray model, and Cox proportional hazards models. Two-sided *P*-values of 0.05 were used to determine statistical significance.

Supplementary Figure 1. Patient selection flowchart.

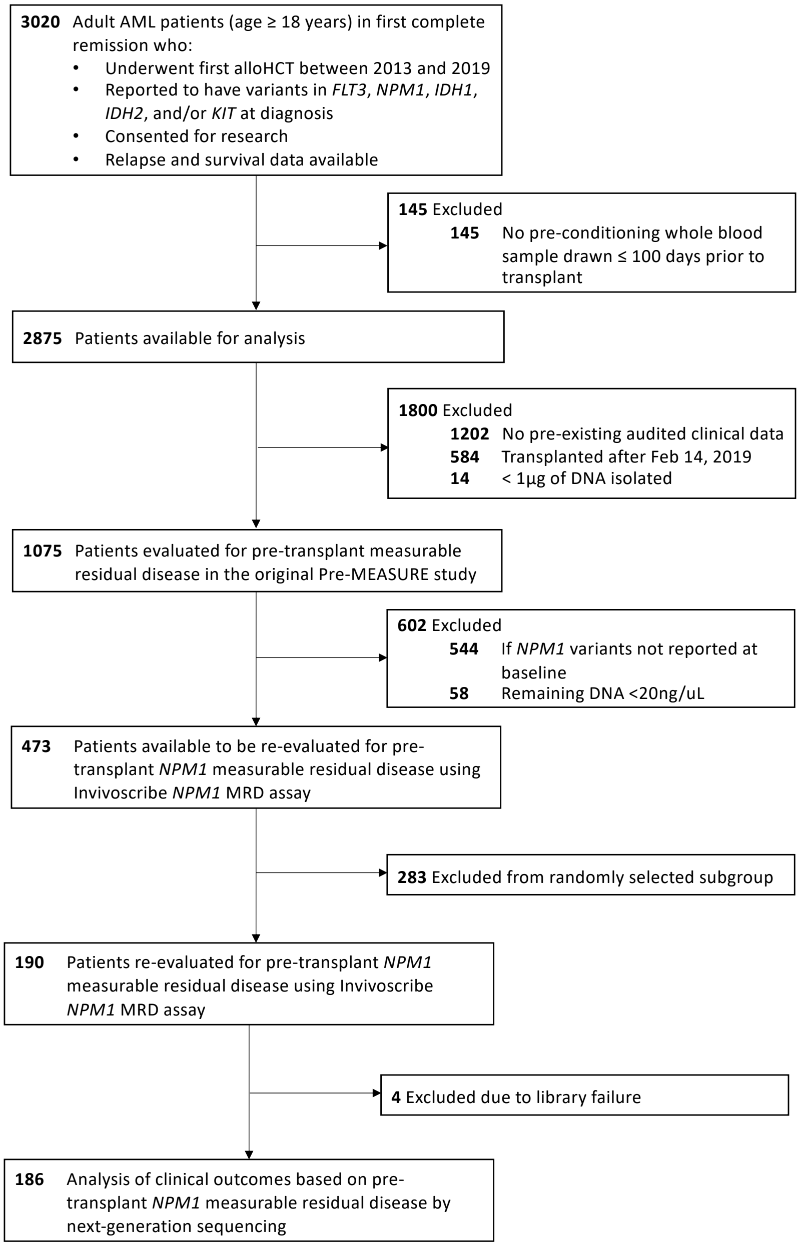

Supplementary Figure 2. Sample size selection. The data from Pre-MEASURE with *NPM1* MRD defined using AMP assay was used for the simulation[1]. We assumed that the new assay would have at least the same sensitivity in identifying high risk patients using relapse endpoint. For each sample size, 1000 random sampling without replacement was performed on the original data, and the hazard ratio (HR) with corresponding *P*-value was calculated and reported for each random subset. Results for the simulation were visualized by plotting -log(p.value) as the x-axis and HR as y-axis, with a vertical line of -log(0.05) as the *P*-value cutoff and a horizontal line of 1 as the HR cutoff, as we expected MRD positive patients had higher risk of relapse compared to those testing negative. The black dots represent the simulation scenarios where HR > 1 and *P*-value < 0.05, while the orange dots represent all other cases. The proportion of the number of black dots among all 1000 simulations was calculated for each sample size. We identified that sample sizes 109, 155, and 190 had the proportion of 0.95, 0.99, and 1. 237 is half of the number of samples with *NPM1* mutations reported at baseline.

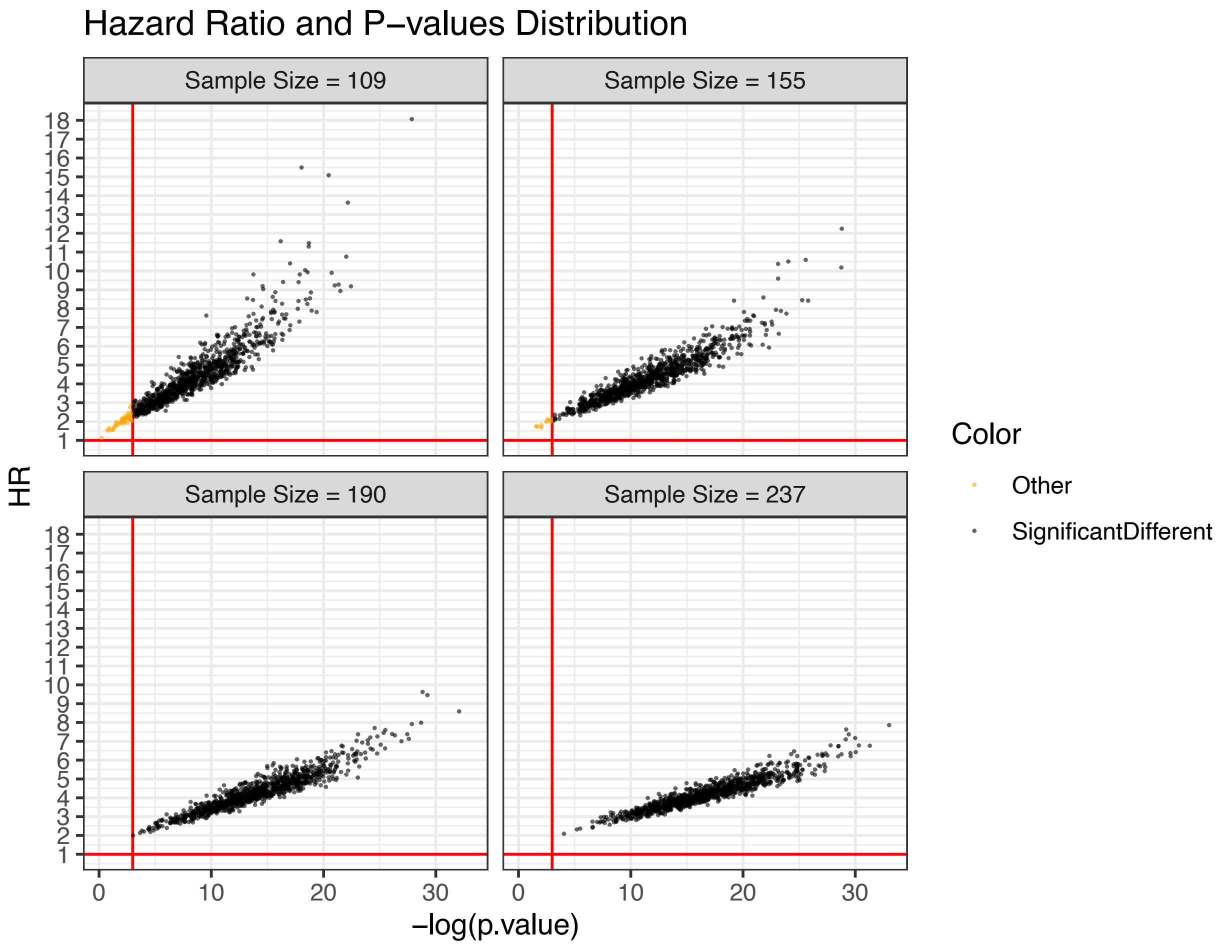

Supplementary Figure 3. Validation of the Invivoscribe *NPM1* MRD assay. (A) Samples, *NPM1* variant type, expected variant allele fraction (VAF), and *NPM1* MRD result for competency standards as evaluated at the Invivoscribe and National Institutes of Health (NIH) laboratories. (B) Graphical representation of VRF of *NPM1* variants as detected by Invivoscribe and NIH laboratories for competency standards versus expected VAF. The Pearson correlation is shown in the graph inset and a value of equivalence line is displayed as a dashed line.

A

		Invivoscribe Lab			NIH Lab		
**Sample Type**	**Targeted VAF**	**VRF**	**Type**	**Result**	**VRF**	**Type**	**Result**
Type A Cell line	1.00E-02	1.05E-02	Type A	Positive	9.68E-03	Type A	Positive
Type A Cell line	1.00E-03	1.19E-03	Type A	Positive	9.37E-04	Type A	Positive
Type A Cell line	5.00E-04	6.36E-04	Type A	Positive	4.82E-04	Type A	Positive
Type A Cell line	1.00E-04	1.38E-04	Type A	Positive	6.93E-05	Type A	Positive
Type A Cell line	5.00E-05	5.01E-05	Type A	Positive	3.56E-05	Type A	Positive
Type A Cell line	1.00E-05	1.30E-05	Type A	Positive	5.89E-06	Type A	Positive
Type A Cell line	1.00E-06	Not Detected	Not Detected	Not Detected	Not Detected	Not Detected	Not Detected
Type B Clinical Positive	5.00E-04	5.09E-04	Type B	Positive	5.10E-04	Type B	Positive
Type B Clinical Positive	1.00E-04	5.36E-05	Type B	Positive	6.33E-05	Type B	Positive
Type B Clinical Positive	5.00E-05	1.18E-05	Type B	Positive	3.31E-05	Type B	Positive
Type B Clinical Positive	1.00E-05	Not Detected	Not Detected	Not Detected	9.68E-06	Type B	Positive
Type D Clinical Positive	5.00E-04	4.21E-04	Type D	Positive	4.12E-04	Type D	Positive
Type D Clinical Positive	1.00E-04	7.58E-05	Type D	Positive	9.05E-05	Type D	Positive
Type D Clinical Positive	5.00E-05	5.03E-05	Type D	Positive	5.11E-05	Type D	Positive
Type D Clinical Positive	1.00E-05	5.57E-06	Type D	Positive	Not Detected	Not Detected	Not Detected
Type Other Clinical Positive	5.00E-04	3.91E-04	Type Other	Positive	3.96E-04	Other	Positive
Type Other Clinical Positive	1.00E-04	1.44E-04	Type Other	Positive	1.28E-04	Other	Positive
Type Other Clinical Positive	5.00E-05	3.86E-05	Type Other	Positive	4.99E-05	Other	Positive
Type Other Clinical Positive	1.00E-05	Not Detected	Not Detected	Not Detected	2.74E-06	Other	Positive
Clinical Negative Pool	n/a	Not Detected	Not Detected	Not Detected	Not Detected	Not Detected	Not Detected
Clinical Negative Pool	n/a	Not Detected	Not Detected	Not Detected	Not Detected	Not Detected	Not Detected
Positive Control	n/a	4.55E-05	Type A	Positive	0.00016	Type A	Positive
Negative Control	n/a	Not Detected	Not Detected	Not Detected	Not Detected	Not Detected	Not Detected
No Template Control	n/a	Undetermined	Undetermined	Undetermined	Undetermined	Undetermined	Undetermined

B

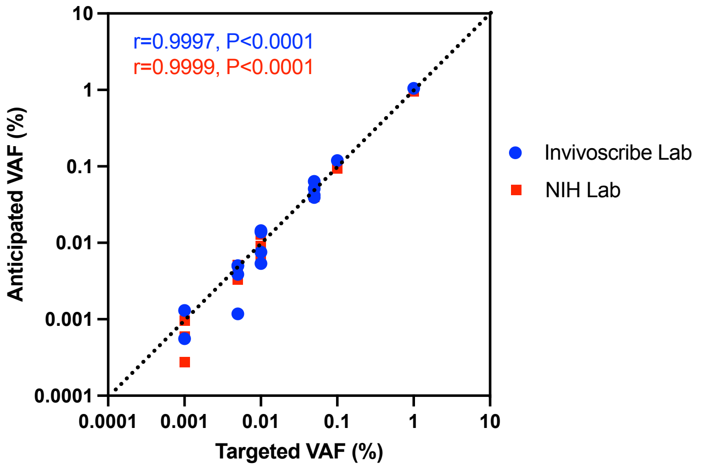

Supplementary Figure 4. VAF comparison between IVS and AMP assays. The variants identified by either assay were included in the figure, and each dot represented the result from one patient, with x-axis as the VAF by IVS and y-axis as the VAF by AMP. The colors were labeled by whether the variants were detected by both assay (red, n=33), and negative cases by both assays were represented by one blue dot (n=109). Dotted lines at 0.01% represented the threshold used previously for the reporting of AMP assay, and the solid red dots were those above the threshold by both assays. The grey line of equality was also plotted.

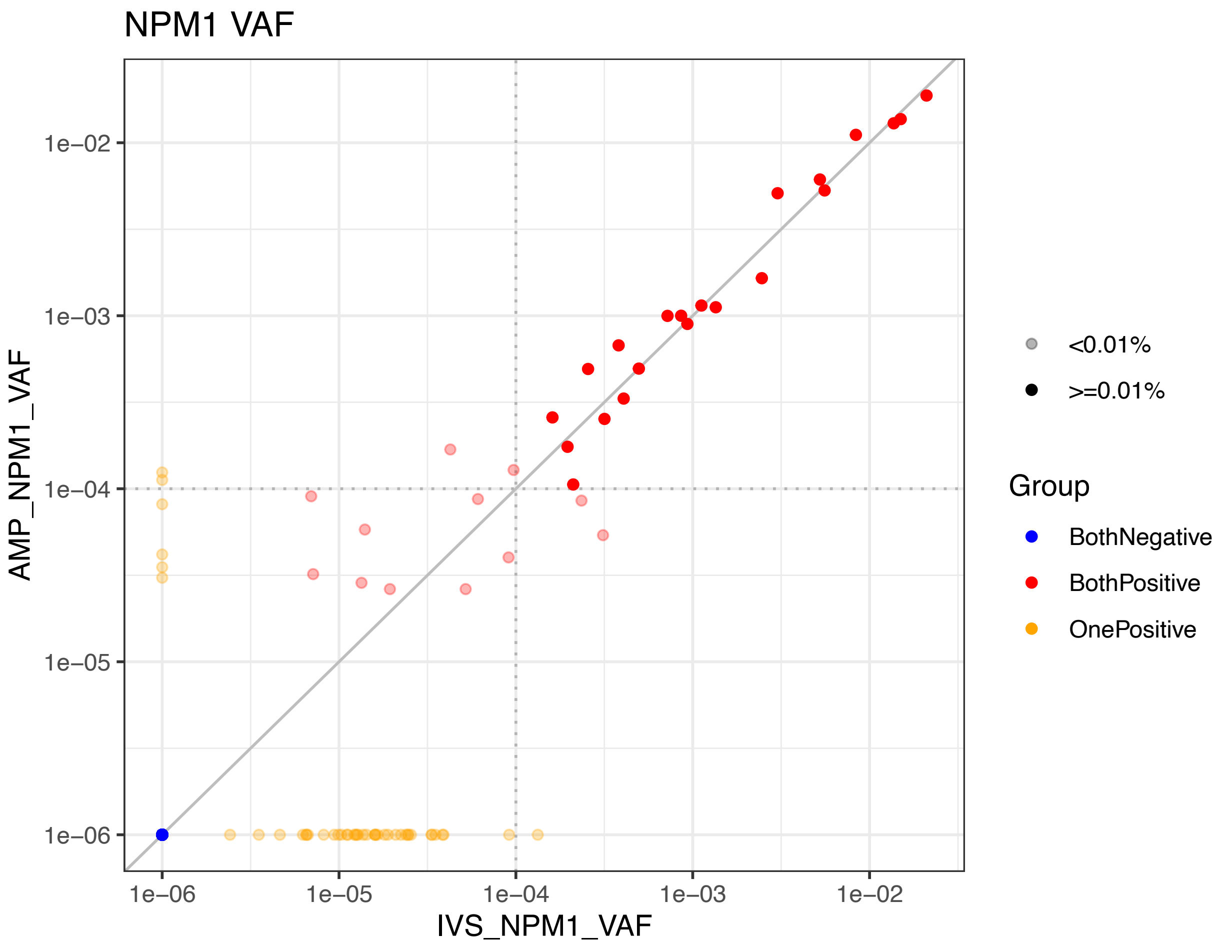

Supplementary Figure 5. *NPM1* MRD status for *NPM1* mutated patients and the association with clinical outcomes after allogeneic hematopoietic cell transplant. Cumulative incidence of non-relapse mortality (NRM, top left) and relapse (top right), relapse-free survival (RFS, bottom left) and overall survival (OS, bottom right) shown at 36 months based on NGS *NPM1* MRD status defined by **(A)** IVS or AMP with a VAF threshold of ≥0.01%; **(B)** IVS with a VAF threshold of >0%; **(C)** IVS with different VAF groups. Point estimates at different time points are shown in the table (far right). Overall P values: Gray's test for non-relapse mortality (NRM) and relapse; log-rank test for relapse-free survival (RFS) and overall survival (OS). P values for pointwise estimations at different time points: z-test. Confidence interval, CI; Probability, prob; Month, mo; Year, yr; pos, positive; neg, negative.

A

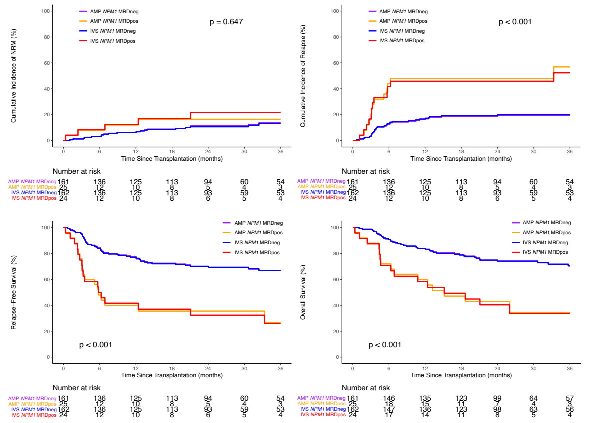

NGS *NPM1* MRD by AMP or IVS, VAF ≥ 0.01%

Outcomes	Prob (95% CI)	Prob (95% CI)	Prob (95% CI)	Prob (95% CI)	p-value
NRM	AMP *NPM1* MRDneg n=161	AMP *NPM1* MRDpos n=25	IVS *NPM1* MRDneg n=162	IVS *NPM1* MRDpos n=24	0.647
3 mo	1(0-3)%	8(0-19)%	1(0-3)%	8(0-20)%	0.403
6 mo	3(0-6)%	8(0-19)%	3(0-6)%	8(0-20)%	0.679
1 yr	6(2-10)%	12(0-25)%	6(2-10)%	13(0-26)%	0.692
2 yr	11(6-16)%	16(1-32)%	11(6-15)%	22(4-39)%	0.611
3 yr	14(8-20)%	16(1-32)%	13(7-19)%	22(4-39)%	0.812
Relapse	AMP *NPM1* MRDneg n=161	AMP *NPM1* MRDpos n=25	IVS *NPM1* MRDneg n=162	IVS *NPM1* MRDpos n=24	<0.001
3 mo	3(0-6)%	16(1-31)%	3(0-6)%	17(1-32)%	0.122
6 mo	12(7-18)%	44(24-64)%	13(8-18)%	42(21-62)%	0.001
1 yr	16(10-22)%	48(28-68)%	17(11-22)%	46(25-66)%	0.001
2 yr	19(13-25)%	48(28-68)%	19(13-25)%	46(25-66)%	0.004
3 yr	19(13-26)%	57(32-81)%	20(14-26)%	52(30-75)%	0.001
RFS	AMP *NPM1* MRDneg n=161	AMP *NPM1* MRDpos n=25	IVS *NPM1* MRDneg n=162	IVS *NPM1* MRDpos n=24	<0.001
3 mo	96(91-98)%	76(54-88)%	96(91-98)%	75(53-88)%	0.015
6 mo	84(78-89)%	48(28-66)%	84(77-89)%	50(29-68)%	<0.001
1 yr	78(70-83)%	40(21-58)%	77(70-83)%	42(22-60)%	<0.001
2 yr	70(62-76)%	36(18-54)%	70(62-77)%	32(15-51)%	<0.001
3 yr	67(59-74)%	27(9-48)%	67(59-74)%	26(10-46)%	<0.001
OS	AMP *NPM1* MRDneg n=161	AMP *NPM1* MRDpos n=25	IVS *NPM1* MRDneg n=162	IVS *NPM1* MRDpos n=24	<0.001
3 mo	99(95-100)%	88(67-96)%	99(95-100)%	88(66-96)%	0.143
6 mo	91(85-94)%	72(50-86)%	91(85-94)%	71(48-85)%	0.038
1 yr	84(77-89)%	60(38-76)%	84(77-89)%	58(36-75)%	0.01
2 yr	75(67-81)%	43(23-61)%	75(68-81)%	40(21-59)%	<0.001
3 yr	70(62-77)%	34(14-55)%	71(62-77)%	34(15-54)%	<0.001

B

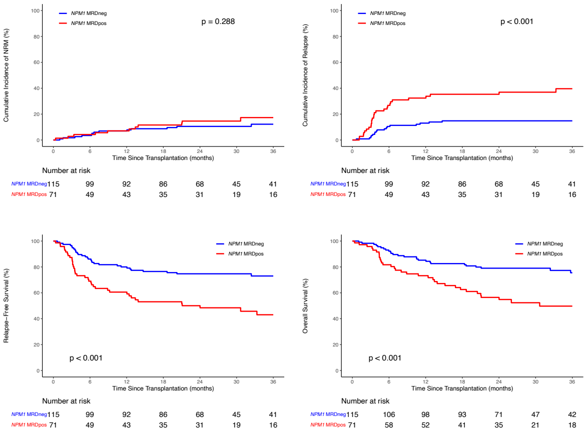

NGS *NPM1* MRD by IVS, VAF > 0

Outcomes	Prob (95% CI)	Prob (95% CI)	p-value
NRM	*NPM1* MRDneg n=115	*NPM1* MRDpos n=71	0.288
3 mo	2(0-4)%	3(0-7)%	0.643
6 mo	3(0-7)%	4(0-9)%	0.8
1 yr	7(2-12)%	7(1-13)%	0.982
2 yr	10(5-16)%	15(6-23)%	0.427
3 yr	12(6-19)%	17(8-27)%	0.391
Relapse	*NPM1* MRDneg n=115	*NPM1* MRDpos n=71	<0.001
3 mo	2(0-4)%	10(3-17)%	0.031
6 mo	10(5-16)%	27(16-37)%	0.007
1 yr	13(7-19)%	32(21-43)%	0.003
2 yr	15(8-21)%	35(24-47)%	0.002
3 yr	15(8-21)%	40(27-52)%	<0.001
RFS	*NPM1* MRDneg n=115	*NPM1* MRDpos n=71	<0.001
3 mo	97(91-99)%	87(77-93)%	0.033
6 mo	86(78-91)%	69(57-78)%	0.007
1 yr	80(71-86)%	61(48-71)%	0.005
2 yr	75(66-82)%	50(38-61)%	<0.001
3 yr	73(63-80)%	43(30-55)%	<0.001
OS	*NPM1* MRDneg n=115	*NPM1* MRDpos n=71	<0.001
3 mo	98(93-100)%	96(87-99)%	0.354
6 mo	92(86-96)%	82(71-89)%	0.045
1 yr	85(77-91)%	73(61-82)%	0.054
2 yr	79(70-85)%	56(44-67)%	0.002
3 yr	75(66-83)%	50(37-62)%	<0.001

C

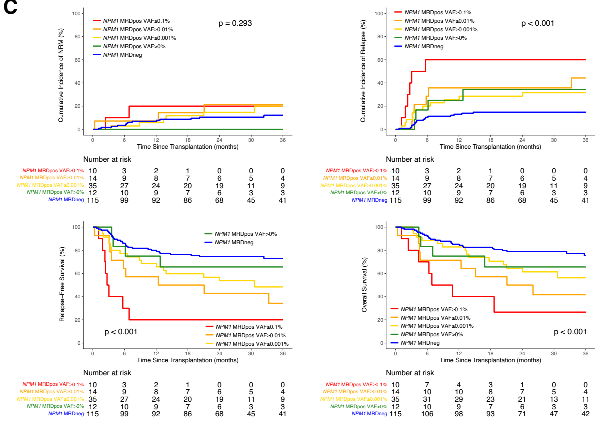

NGS *NPM1* MRD by IVS, Different VAF Groups

Outcomes	Prob (95% CI)	Prob (95% CI)	Prob (95% CI)	Prob (95% CI)	Prob (95% CI)	p-value
NRM	*NPM1* MRDpos VAF≥0.1% n=10	*NPM1* MRDpos VAF≥0.01% n=14	*NPM1* MRDpos VAF≥0.001% n=35	*NPM1* MRDpos VAF>0% n=12	*NPM1* MRDneg n=115	0.293
3 mo	10(0-30)%	7(0-21)%	0(0-0)%	0(0-0)%	2(0-4)%	NA
6 mo	10(0-30)%	7(0-21)%	3(0-8)%	0(0-0)%	3(0-7)%	0.133
1 yr	20(0-48)%	7(0-21)%	6(0-14)%	0(0-0)%	7(2-12)%	0.009
2 yr	20(0-48)%	21(0-44)%	15(3-27)%	0(0-0)%	10(5-16)%	<0.001
3 yr	20(0-48)%	21(0-44)%	20(4-36)%	0(0-0)%	12(6-19)%	<0.001
Relapse	*NPM1* MRDpos VAF≥0.1% n=10	*NPM1* MRDpos VAF≥0.01% n=14	*NPM1* MRDpos VAF≥0.001% n=35	*NPM1* MRDpos VAF>0% n=12	*NPM1* MRDneg n=115	<0.001
3 mo	40(7-73)%	0(0-0)%	9(0-18)%	0(0-0)%	2(0-4)%	NA
6 mo	60(26-94)%	29(4-53)%	20(7-33)%	17(0-39)%	10(5-16)%	0.028
1 yr	60(26-94)%	36(9-62)%	26(11-40)%	25(0-51)%	13(7-19)%	0.022
2 yr	60(26-94)%	36(9-62)%	29(13-44)%	34(5-63)%	15(8-21)%	0.023
3 yr	60(26-94)%	44(16-73)%	32(16-47)%	34(5-63)%	15(8-21)%	0.009
RFS	*NPM1* MRDpos VAF≥0.1% n=10	*NPM1* MRDpos VAF≥0.01% n=14	*NPM1* MRDpos VAF≥0.001% n=35	*NPM1* MRDpos VAF>0% n=12	*NPM1* MRDneg n=115	<0.001
3 mo	50(18-75)%	93(59-99)%	91(76-97)%	100%	97(91-99)%	<0.001
6 mo	30(7-58)%	64(34-83)%	77(59-88)%	83(48-96)%	86(78-91)%	0.002
1 yr	20(3-47)%	57(28-78)%	69(50-81)%	75(41-91)%	80(71-86)%	<0.001
2 yr	20(3-47)%	43(18-66)%	57(39-71)%	66(32-86)%	75(66-82)%	<0.001
3 yr	20(3-47)%	34(12-59)%	48(30-65)%	66(32-86)%	73(63-80)%	<0.001
OS	*NPM1* MRDpos VAF≥0.1% n=10	*NPM1* MRDpos VAF≥0.01% n=14	*NPM1* MRDpos VAF≥0.001% n=35	*NPM1* MRDpos VAF>0% n=12	*NPM1* MRDneg n=115	<0.001
3 mo	80(41-95)%	93(59-99)%	100%	100%	98(93-100)%	NA
6 mo	70(33-89)%	71(41-88)%	89(72-96)%	83(48-96)%	92(86-96)%	0.242
1 yr	40(12-67)%	71(41-88)%	83(66-92)%	75(41-91)%	85(77-91)%	0.052
2 yr	27(5-56)%	50(23-72)%	64(46-78)%	66(32-86)%	79(70-85)%	0.003
3 yr	27(5-56)%	42(16-65)%	56(37-72)%	66(32-86)%	75(66-83)%	0.003

Supplementary Figure 6. Multivariable regression analyses. Multivariable competing-risks regression for relapse (top) and Cox regression analysis for OS (bottom) for residual *NPM1* variants grouped by residual disease burden level. Models were selected stepwise using MRD status and baseline clinical characteristics. VAF, variant allele fraction; KPS, Karnofsky performance score.

Multivariable Competing Risks Regression for Relapse

Measure	Hazard Ratio	P value
NPM1 MRDpos		
VAF ≥ 0.1%	5.67 (1.83–17.54)	0.003
VAF ≥ 0.01%	4.39 (2.11–9.14)	<0.001
VAF ≥ 0.001%	2.52 (1.21–5.22)	0.013
VAF > 0%	2.32 (0.82–6.58)	0.11
KPS ≥ 90	1.95 (1.05–3.61)	0.034

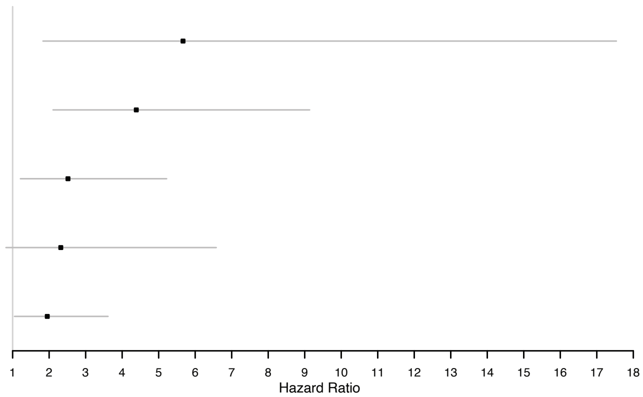

Multivariable Cox Regression for Overall Survival

Measure	Hazard Ratio	P value
NPM1 MRDpos		
VAF ≥ 0.1%	6.44 (2.75–15.07)	<0.001
VAF ≥ 0.01%	4.01 (1.93–8.34)	<0.001
VAF ≥ 0.001%	2.37 (1.28–4.36)	0.006
VAF > 0%	1.76 (0.61–5.06)	0.291

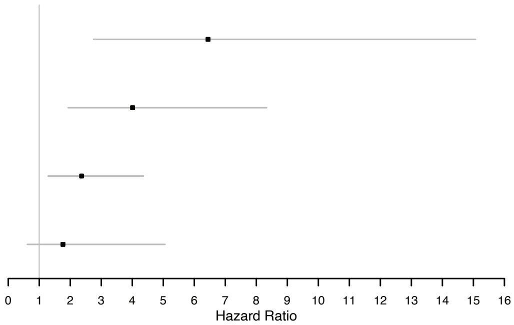

Supplementary Figure 7. *NPM1* MRD status for *NPM1* mutated patients by IVS using a VAF threshold of >0% and the association with clinical outcomes after allogeneic hematopoietic cell transplant considering baseline *FLT3*-ITD mutations. (A) Patients without *FLT3*-ITD mutation at baseline; **(B)** Patients with *FLT3*-ITD mutation at baseline. Point estimates at different time points are shown in the table (far right). Overall P values: Gray's test for non-relapse mortality (NRM) and relapse; log-rank test for relapse-free survival (RFS) and overall survival (OS). P values for pointwise estimations at different time points: z-test. Confidence interval, CI; Probability, prob; Month, mo; Year, yr; pos, positive; neg, negative.

A

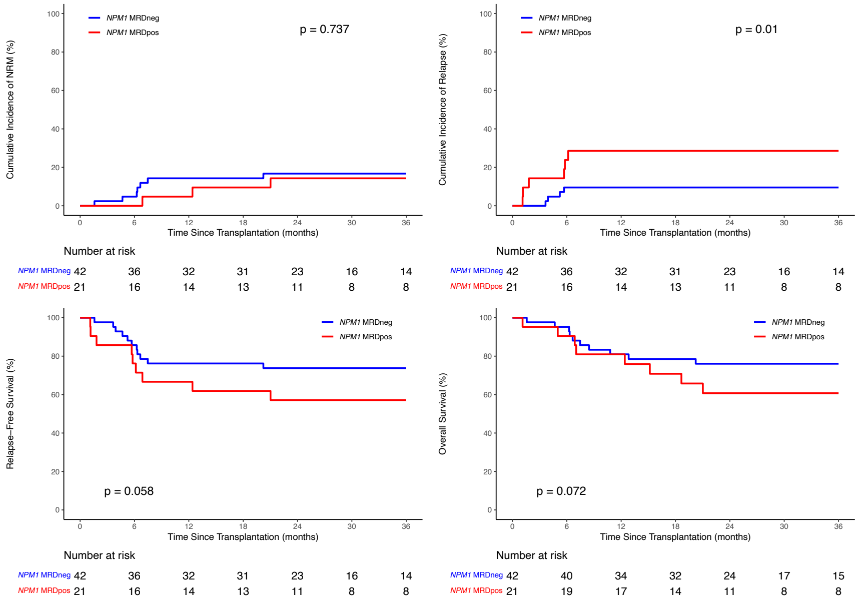

NGS *NPM1* MRD by IVS
for Patients without Baseline *FLT3*-ITD

Outcomes	Prob (95% CI)	Prob (95% CI)	p-value
NRM	*NPM1* MRDneg n=42	*NPM1* MRDpos n=21	0.737
3 mo	2(0-7)%	0(0-0)%	0.317
6 mo	5(0-11)%	0(0-0)%	0.152
1 yr	14(4-25)%	5(0-14)%	0.191
2 yr	17(5-28)%	14(0-30)%	0.803
3 yr	17(5-28)%	14(0-30)%	0.803
Relapse	*NPM1* MRDneg n=42	*NPM1* MRDpos n=21	0.01
3 mo	0(0-0)%	14(0-30)%	0.068
6 mo	10(1-19)%	24(5-43)%	0.178
1 yr	10(1-19)%	29(9-48)%	0.087
2 yr	10(1-19)%	29(9-48)%	0.087
3 yr	10(1-19)%	29(9-48)%	0.087
RFS	*NPM1* MRDneg n=42	*NPM1* MRDpos n=21	0.058
3 mo	98(84-100)%	86(62-95)%	0.136
6 mo	86(71-93)%	76(52-89)%	0.376
1 yr	76(60-86)%	67(43-83)%	0.435
2 yr	74(58-85)%	57(34-75)%	0.194
3 yr	74(58-85)%	57(34-75)%	0.194
OS	*NPM1* MRDneg n=42	*NPM1* MRDpos n=21	0.072
3 mo	98(84-100)%	95(71-99)%	0.648
6 mo	95(82-99)%	90(67-98)%	0.508
1 yr	81(66-90)%	81(57-92)%	1
2 yr	76(60-86)%	61(36-78)%	0.228
3 yr	76(60-86)%	61(36-78)%	0.228

B

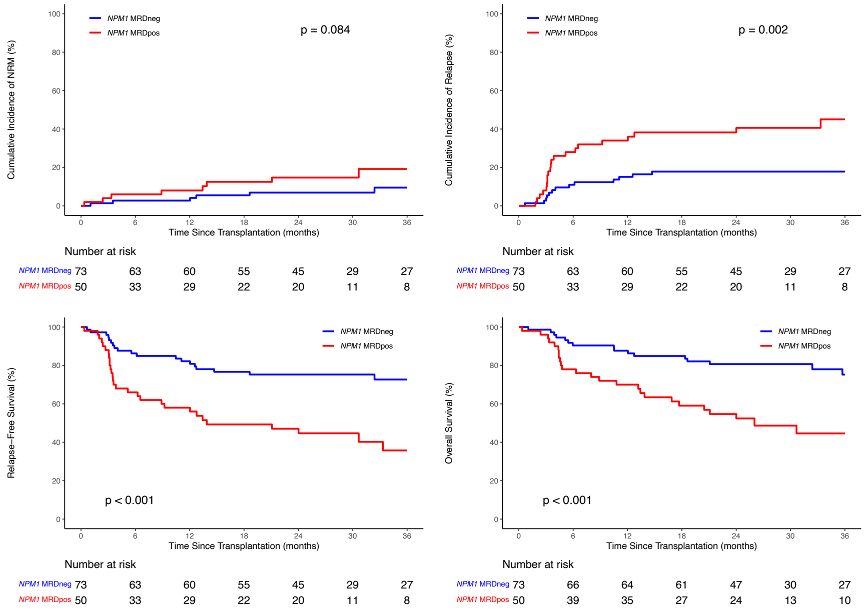

NGS *NPM1* MRD by IVS
for Patients with Baseline *FLT3*-ITD

Outcomes	Prob (95% CI)	Prob (95% CI)	p-value
NRM	*NPM1* MRDneg n=73	*NPM1* MRDpos n=50	0.084
3 mo	1(0-4)%	4(0-9)%	0.399
6 mo	3(0-7)%	6(0-13)%	0.404
1 yr	3(0-7)%	8(0-16)%	0.225
2 yr	7(1-13)%	15(4-25)%	0.195
3 yr	9(2-17)%	19(6-32)%	0.212
Relapse	*NPM1* MRDneg n=73	*NPM1* MRDpos n=50	0.002
3 mo	3(0-7)%	8(0-16)%	0.224
6 mo	11(4-18)%	28(15-41)%	0.021
1 yr	15(7-23)%	34(21-47)%	0.018
2 yr	18(9-27)%	38(24-52)%	0.014
3 yr	18(9-27)%	45(29-61)%	0.003
RFS	*NPM1* MRDneg n=73	*NPM1* MRDpos n=50	<0.001
3 mo	96(88-99)%	88(75-94)%	0.125
6 mo	86(76-92)%	66(51-77)%	0.009
1 yr	82(71-89)%	58(43-70)%	0.004
2 yr	75(64-84)%	47(33-60)%	0.001
3 yr	73(60-82)%	36(21-51)%	<0.001
OS	*NPM1* MRDneg n=73	*NPM1* MRDpos n=50	<0.001
3 mo	99(91-100)%	96(85-99)%	0.394
6 mo	90(81-95)%	78(64-87)%	0.068
1 yr	88(78-93)%	70(55-81)%	0.019
2 yr	81(70-88)%	55(40-67)%	0.002
3 yr	75(62-84)%	45(29-59)%	0.002

Supplementary Figure 8. NGS MRD status for *NPM1* and *FLT3*-ITD mutated patients and the association with clinical outcomes after allogeneic hematopoietic cell transplant. MRD status was defined using *NPM1* only (blue/red), *FLT3*-ITD only (green/yellow), or *NPM1* and/or *FLT3*-ITD (purple/orange). The cohort had 123 patients in total, and each patient was assigned to three groups based on different MRD definitions. **(A)** The subset of patients reported using IVS results; **(B)** The full cohort reported using AMP results. Point estimates at different time points are shown in the table (far right). Overall P values: Gray's test for non-relapse mortality (NRM) and relapse; log-rank test for relapse-free survival (RFS) and overall survival (OS). P values for pointwise estimations at different time points: z-test. Confidence interval, CI; Probability, prob; Month, mo; Year, yr; pos, positive; neg, negative.

A

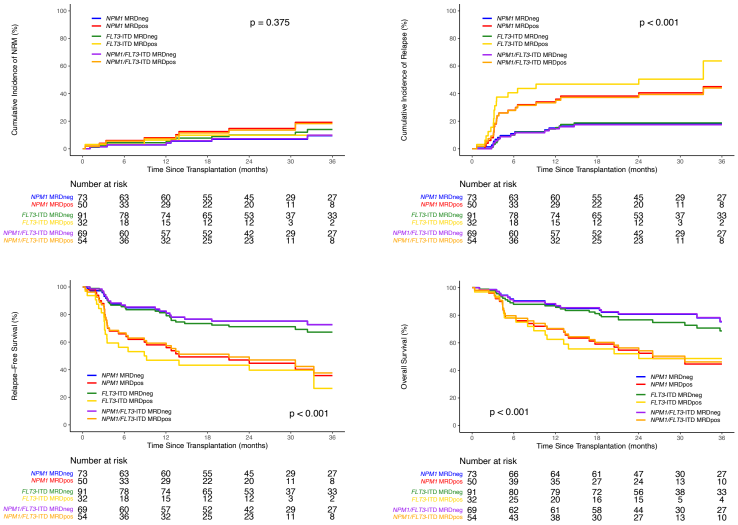

NGS MRD by IVS *NPM1* and *FLT3*-ITD

Outcomes	Prob (95% CI)	Prob (95% CI)	Prob (95% CI)	Prob (95% CI)	Prob (95% CI)	Prob (95% CI)	p-value
NRM	*NPM1* MRDneg n=73	*NPM1* MRDpos n=50	*FLT3*-ITD MRDneg n=91	*FLT3*-ITD MRDpos n=32	*NPM1/FLT3*-ITD MRDneg n=69	*NPM1/FLT3*-ITD MRDpos n=54	0.375
3 mo	1(0-4)%	4(0-9)%	2(0-5)%	3(0-9)%	1(0-4)%	4(0-9)%	0.92
6 mo	3(0-7)%	6(0-13)%	4(0-9)%	3(0-9)%	3(0-7)%	6(0-12)%	0.93
1 yr	3(0-7)%	8(0-16)%	4(0-9)%	6(0-15)%	3(0-7)%	7(0-14)%	0.707
2 yr	7(1-13)%	15(4-25)%	10(4-16)%	10(0-21)%	7(1-14)%	14(4-23)%	0.709
3 yr	9(2-17)%	19(6-32)%	14(6-22)%	10(0-21)%	10(2-18)%	18(5-31)%	0.684
Relapse	*NPM1* MRDneg n=73	*NPM1* MRDpos n=50	*FLT3*-ITD MRDneg n=91	*FLT3*-ITD MRDpos n=32	*NPM1/FLT3*-ITD MRDneg n=69	*NPM1/FLT3*-ITD MRDpos n=54	<0.001
3 mo	3(0-7)%	8(0-16)%	1(0-3)%	16(3-28)%	1(0-4)%	9(1-17)%	0.052
6 mo	11(4-18)%	28(15-41)%	10(4-16)%	41(23-58)%	10(3-17)%	28(16-40)%	<0.001
1 yr	15(7-23)%	34(21-47)%	14(7-22)%	47(29-65)%	14(6-23)%	33(21-46)%	<0.001
2 yr	18(9-27)%	38(24-52)%	19(11-27)%	47(29-65)%	17(8-26)%	37(24-50)%	0.001
3 yr	18(9-27)%	45(29-61)%	19(11-27)%	64(34-93)%	17(8-26)%	44(29-60)%	<0.001
RFS	*NPM1* MRDneg n=73	*NPM1* MRDpos n=50	*FLT3*-ITD MRDneg n=91	*FLT3*-ITD MRDpos n=32	*NPM1/FLT3*-ITD MRDneg n=69	*NPM1/FLT3*-ITD MRDpos n=54	p < 0.001
3 mo	96(88-99)%	88(75-94)%	97(90-99)%	81(63-91)%	97(89-99)%	87(75-94)%	0.045
6 mo	86(76-92)%	66(51-77)%	86(77-91)%	56(38-71)%	87(76-93)%	67(52-78)%	<0.001
1 yr	82(71-89)%	58(43-70)%	81(72-88)%	47(29-63)%	83(71-90)%	59(45-71)%	<0.001
2 yr	75(64-84)%	47(33-60)%	71(61-79)%	43(26-60)%	75(63-84)%	49(35-62)%	<0.001
3 yr	73(60-82)%	36(21-51)%	67(56-76)%	26(7-51)%	73(60-82)%	38(22-53)%	<0.001
OS	*NPM1* MRDneg n=73	*NPM1* MRDpos n=50	*FLT3*-ITD MRDneg n=91	*FLT3*-ITD MRDpos n=32	*NPM1/FLT3*-ITD MRDneg n=69	*NPM1/FLT3*-ITD MRDpos n=54	p < 0.001
3 mo	99(91-100)%	96(85-99)%	98(91-99)%	97(80-100)%	99(90-100)%	96(86-99)%	0.917
6 mo	90(81-95)%	78(64-87)%	88(79-93)%	78(60-89)%	90(80-95)%	80(66-88)%	0.2
1 yr	88(78-93)%	70(55-81)%	87(78-92)%	62(44-77)%	88(78-94)%	70(56-81)%	0.002
2 yr	81(70-88)%	55(40-67)%	77(66-84)%	52(33-68)%	81(70-89)%	56(42-68)%	<0.001
3 yr	75(62-84)%	45(29-59)%	69(56-78)%	49(30-65)%	76(62-85)%	46(30-61)%	<0.001

B

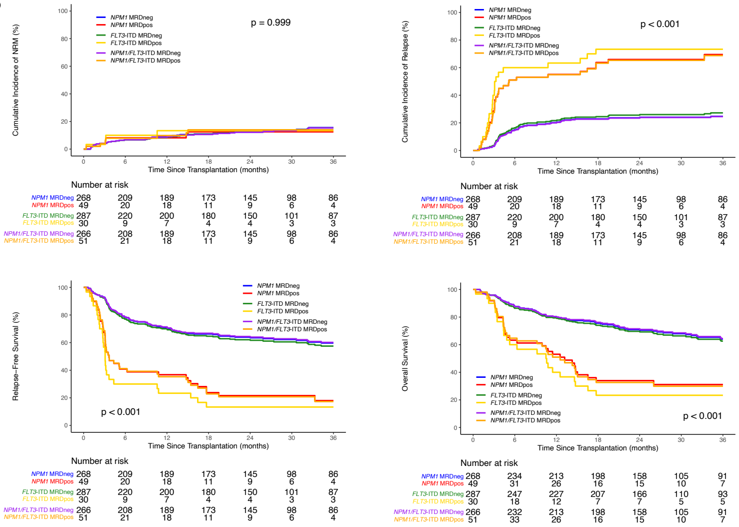

NGS MRD by AMP *NPM1* and *FLT3*-ITD

Outcomes	Prob (95% CI)	Prob (95% CI)	Prob (95% CI)	Prob (95% CI)	Prob (95% CI)	Prob (95% CI)	p-value
NRM	*NPM1* MRDneg n=268	*NPM1* MRDpos n=49	*FLT3*-ITD MRDneg n=287	*FLT3*-ITD MRDpos n=30	*NPM1/FLT3*-ITD MRDneg n=266	*NPM1/FLT3*-ITD MRDpos n=51	0.999
3 mo	4(2-6)%	4(0-10)%	4(2-7)%	3(0-10)%	4(2-7)%	4(0-9)%	1
6 mo	7(4-10)%	8(0-16)%	7(4-10)%	10(0-21)%	7(4-10)%	8(0-15)%	0.991
1 yr	9(6-12)%	8(0-16)%	8(5-12)%	13(1-26)%	9(5-12)%	10(2-18)%	0.985
2 yr	12(8-16)%	12(3-22)%	12(8-16)%	13(1-26)%	12(8-16)%	14(4-24)%	1
3 yr	16(11-20)%	12(3-22)%	15(11-20)%	13(1-26)%	15(11-20)%	14(4-24)%	0.993
Relapse	*NPM1* MRDneg n=268	*NPM1* MRDpos n=49	*FLT3*-ITD MRDneg n=287	*FLT3*-ITD MRDpos n=30	*NPM1/FLT3*-ITD MRDneg n=266	*NPM1/FLT3*-ITD MRDpos n=51	<0.001
3 mo	3(1-5)%	29(16-41)%	3(1-6)%	40(22-58)%	3(1-5)%	27(15-40)%	<0.001
6 mo	15(11-19)%	51(37-65)%	16(12-21)%	60(42-78)%	15(10-19)%	51(37-65)%	<0.001
1 yr	20(15-25)%	55(41-69)%	22(17-26)%	63(45-81)%	20(15-25)%	55(41-69)%	<0.001
2 yr	24(19-29)%	66(52-80)%	26(21-31)%	73(57-90)%	23(18-29)%	65(52-79)%	<0.001
3 yr	25(19-30)%	70(55-84)%	27(22-33)%	73(57-90)%	25(19-30)%	69(55-83)%	<0.001
RFS	*NPM1* MRDneg n=268	*NPM1* MRDpos n=49	*FLT3*-ITD MRDneg n=287	*FLT3*-ITD MRDpos n=30	*NPM1/FLT3*-ITD MRDneg n=266	*NPM1/FLT3*-ITD MRDpos n=51	p < 0.001
3 mo	93(89-95)%	67(52-79)%	92(89-95)%	57(37-72)%	93(89-95)%	69(54-79)%	<0.001
6 mo	78(73-83)%	41(27-54)%	77(72-81)%	30(15-47)%	79(73-83)%	41(28-54)%	<0.001
1 yr	71(65-76)%	37(24-50)%	70(64-75)%	23(10-39)%	71(66-76)%	35(23-48)%	<0.001
2 yr	64(58-69)%	22(11-34)%	62(56-67)%	13(4-28)%	64(58-70)%	21(11-33)%	<0.001
3 yr	60(53-66)%	18(8-31)%	57(51-63)%	13(4-28)%	60(54-66)%	17(8-30)%	<0.001
OS	*NPM1* MRDneg n=268	*NPM1* MRDpos n=49	*FLT3*-ITD MRDneg n=287	*FLT3*-ITD MRDpos n=30	*NPM1/FLT3*-ITD MRDneg n=266	*NPM1/FLT3*-ITD MRDpos n=51	p < 0.001
3 mo	96(93-98)%	92(80-97)%	96(93-98)%	90(72-97)%	96(93-98)%	92(80-97)%	0.709
6 mo	88(83-91)%	63(48-75)%	86(82-90)%	60(40-75)%	88(83-91)%	65(50-76)%	<0.001
1 yr	80(74-84)%	53(38-66)%	79(74-84)%	40(23-57)%	80(75-85)%	51(37-64)%	<0.001
2 yr	71(65-76)%	34(21-47)%	70(64-75)%	23(10-39)%	71(66-77)%	33(20-46)%	<0.001
3 yr	64(57-70)%	31(19-45)%	63(56-68)%	23(10-39)%	64(58-70)%	30(18-43)%	<0.001

Supplementary Figure 9. NGS MRD status for all *NPM1* mutated patients from the PreMEASURE study and the association with clinical outcomes after allogeneic hematopoietic cell transplant stratified by conditioning intensity. Point estimates at different time points are shown in the table (far right). Overall P values: Gray's test for non-relapse mortality (NRM) and relapse; log-rank test for relapse-free survival (RFS) and overall survival (OS). P values for pointwise estimations at different time points: z-test. Confidence interval, CI; Probability, prob; Month, mo; Year, yr; pos, positive; neg, negative; RIC, reduced-intensity conditioning without melphalan (mel); NMA, nonmyeloablative conditioning; MAC, myeloablative conditioning.

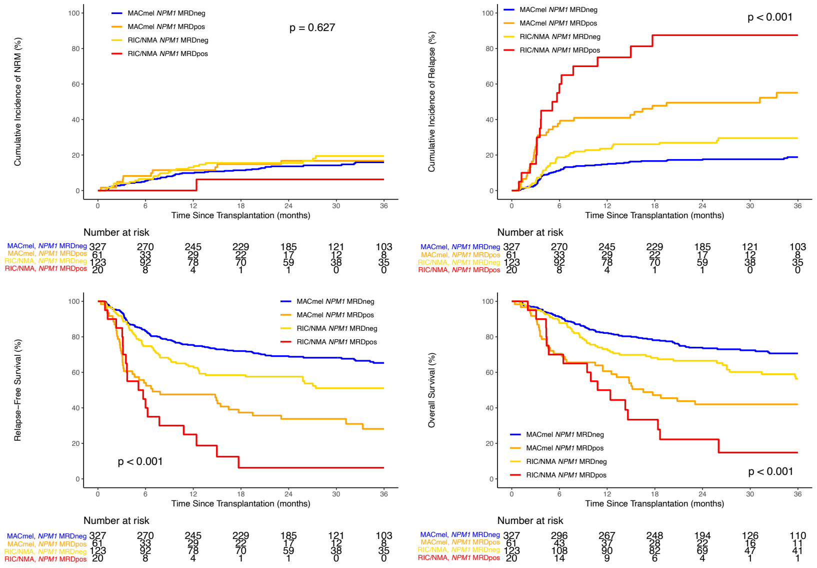

NGS *NPM1* MRD by Conditioning Intensity

Outcomes	Prob (95% CI)	Prob (95% CI)	Prob (95% CI)	Prob (95% CI)	p-value
NRM	MACmel, *NPM1* MRDneg n=327	MACmel *NPM1* MRDpos n=61	RIC/NMA *NPM1* MRDneg n=123	RIC/NMA *NPM1* MRDpos n=20	0.627
3 mo	3(1-5)%	5(0-10)%	4(1-8)%	0(0-0)%	<0.001
6 mo	6(3-8)%	8(1-15)%	7(2-11)%	0(0-0)%	<0.001
1 yr	10(7-13)%	11(3-20)%	13(7-19)%	0(0-0)%	<0.001
2 yr	14(10-17)%	17(7-26)%	15(9-22)%	6(0-20)%	0.6
3 yr	16(12-20)%	17(7-26)%	19(12-27)%	6(0-20)%	0.408
Relapse	MACmel, *NPM1* MRDneg n=327	MACmel *NPM1* MRDpos n=61	RIC/NMA *NPM1* MRDneg n=123	RIC/NMA *NPM1* MRDpos n=20	<0.001
3 mo	3(1-5)%	25(14-36)%	5(1-9)%	15(0-31)%	0.001
6 mo	11(8-15)%	38(25-50)%	19(12-26)%	60(38-82)%	<0.001
1 yr	15(11-19)%	41(28-53)%	24(16-31)%	75(55-95)%	<0.001
2 yr	17(13-21)%	49(37-62)%	27(19-35)%	87(69-100)%	<0.001
3 yr	19(14-23)%	55(41-69)%	30(21-38)%	87(69-100)%	<0.001
RFS	MACmel, *NPM1* MRDneg n=327	MACmel *NPM1* MRDpos n=61	RIC/NMA *NPM1* MRDneg n=123	RIC/NMA *NPM1* MRDpos n=20	<0.001
3 mo	94(90-96)%	70(57-80)%	91(84-95)%	85(60-95)%	0.001
6 mo	83(79-87)%	54(41-66)%	75(66-82)%	40(19-60)%	<0.001
1 yr	75(70-80)%	48(35-59)%	63(54-71)%	25(9-45)%	<0.001
2 yr	69(64-74)%	34(22-46)%	58(48-66)%	6(0-24)%	<0.001
3 yr	65(59-71)%	28(17-41)%	51(41-60)%	6(0-24)%	<0.001
OS	MACmel, *NPM1* MRDneg n=327	MACmel *NPM1* MRDpos n=61	RIC/NMA *NPM1* MRDneg n=123	RIC/NMA *NPM1* MRDpos n=20	<0.001
3 mo	97(94-98)%	92(81-97)%	95(89-98)%	95(69-99)%	0.556
6 mo	91(87-94)%	70(57-80)%	88(81-92)%	70(45-85)%	0.001
1 yr	82(78-86)%	61(47-72)%	73(64-80)%	50(27-69)%	<0.001
2 yr	74(69-78)%	42(29-54)%	67(57-74)%	22(7-43)%	<0.001
3 yr	71(65-76)%	42(29-54)%	56(46-65)%	15(3-36)%	<0.001

Supplementary Table 1. *NPM1*-mutated AML patient baseline clinical characteristics.

Variable	All *NPM1* Baseline Patients (n=531)	Available for Re-Analysis (n=473)	Analyzed (n=186)
**Age Group**			
<60	291 (55%)	253 (53%)	98 (53%)
≥60	240 (45%)	220 (47%)	88 (47%)
**Sex**			
Female	305 (57%)	273 (58%)	109 (59%)
Male	226 (43%)	200 (42%)	77 (41%)
**Conditioning Intensity**			
MAC or Mel	388 (73%)	346 (73%)	144 (77%)
RIC or NMA	143 (27%)	127 (27%)	42 (23%)
**Graft Type**			
Bone Marrow	84 (16%)	74 (16%)	20 (11%)
Cord Blood	73 (14%)	65 (14%)	25 (13%)
Peripheral Blood	374 (70%)	334 (71%)	141 (76%)
**Donor Type**			
Cord Blood	73 (14%)	65 (14%)	25 (13%)
Haploidentical Related	41 (8%)	37 (8%)	12 (6%)
HLA-identical sibling	63 (12%)	57 (12%)	26 (14%)
Matched Unrelated	325 (61%)	287 (61%)	114 (61%)
Mismatched	29 (5%)	27 (6%)	9 (5%)
**HCT-Comorbidity Index**			
0	106 (20%)	98 (21%)	33 (18%)
1,2	172 (33%)	154 (33%)	69 (37%)
3+	247 (47%)	217 (46%)	84 (45%)
**Karnofsky Score**			
<90	233 (44%)	208 (44%)	83 (45%)
>=90	294 (56%)	262 (56%)	101 (55%)
**ATG Usage**			
No	392 (74%)	349 (74%)	136 (73%)
Yes	137 (26%)	122 (26%)	50 (27%)
**Race**			
Caucasian	463 (89%)	412 (89%)	156 (86%)
Other	57 (11%)	51 (11%)	25 (14%)
**ELN Risk Group**			
Adverse	48 (9%)	43 (9%)	10 (5%)
Favorable	178 (34%)	160 (34%)	55 (30%)
Intermediate	303 (57%)	268 (57%)	120 (65%)
**AML Type**			
De novo	471 (89%)	422 (89%)	163 (88%)
Therapy-related	18 (3%)	15 (3%)	5 (3%)
Transformed MDS/MPN	42 (8%)	36 (8%)	18 (10%)
**Transplant Year**			
2013	21 (4%)	19 (4%)	3 (2%)
2014	70 (13%)	68 (14%)	26 (14%)
2015	54 (10%)	48 (10%)	23 (12%)
2016	59 (11%)	44 (9%)	17 (9%)
2017	35 (7%)	31 (7%)	15 (8%)
2018	258 (49%)	232 (49%)	91 (49%)
2019	33 (6%)	30 (6%)	11 (6%)

Supplementary Table 2. Residual *NPM1* and *FLT3*-ITD Variants Detected in the Blood of AML Patients Prior to Transplant.

Patient ID	IVS NPM1 MRD Result	IVS NPM1 Type	IVS NPM1 HGVSc	IVS NPM1 HGVSp	IVS NPM1 VAF (%)	AMP NPM1 MRD Result	AMP NPM1 Type	AMP NPM1 HGVSc	AMP NPM1 HGVSp	AMP NPM1 VAF (%)	FLT3-ITD Baseline	IVS FLT3-ITD MRD Result	IVS FLT3-ITD VAF (%)
CH012058	Positive	Type A	NM_002520.7:c.860_863dup	NP_002511.1::p.W288Cfs*12	0.00385	Not Detected	NA	NA	NA	NA	Positive	Positive	0.00152
CH012068	Positive	Type A	NM_002520.7:c.860_863dup	NP_002511.1::p.W288Cfs*12	0.07200	Positive	Type A	NM_002520.6:c.860_863dup	NP_002511.1:p.Trp288CysfsTer12	0.09981	Negative	Not Tested	NA
CH012131	Positive	Type A	NM_002520.7:c.860_863dup	NP_002511.1::p.W288Cfs*12	0.01960	Positive	Type A	NM_002520.6:c.860_863dup	NP_002511.1:p.Trp288CysfsTer12	0.01748	Positive	Not Detected	0
CH012135	Positive	Type A	NM_002520.7:c.860_863dup	NP_002511.1::p.W288Cfs*12	0.00067	Not Detected	NA	NA	NA	NA	Positive	Not Detected	0
CH012172	Positive	Type A	NM_002520.7:c.860_863dup	NP_002511.1::p.W288Cfs*12	0.00103	Not Detected	NA	NA	NA	NA	Positive	Not Detected	0
CH012200	Positive	Type A	NM_002520.7:c.860_863dup	NP_002511.1::p.W288Cfs*12	0.00160	Not Detected	NA	NA	NA	NA	Positive	Not Detected	0
CH012221	Positive	Type A	NM_002520.7:c.860_863dup	NP_002511.1::p.W288Cfs*12	0.03170	Positive	Type A	NM_002520.6:c.860_863dup	NP_002511.1:p.Trp288CysfsTer12	0.02533	Positive	Positive	0.00783
CH012285	Positive	Type A	NM_002520.7:c.860_863dup	NP_002511.1::p.W288Cfs*12	0.03110	Positive	Type A	NM_002520.6:c.860_863dup	NP_002511.1:p.Trp288CysfsTer12	0.00539	Positive	Not Detected	0
CH012303	Positive	Type A	NM_002520.7:c.860_863dup	NP_002511.1::p.W288Cfs*12	0.00390	Not Detected	NA	NA	NA	NA	Negative	Not Tested	NA
CH012325	Positive	Type D	NM_002520.7:c.863_864insCCTG	NP_002511.1::p.W288Cfs*12	0.00134	Positive	Type D	NM_002520.6:c.863_864insCCTG	NP_002511.1:p.Trp288CysfsTer12	0.00286	Positive	Positive	0.00173
CH012370	Positive	Type A	NM_002520.7:c.860_863dup	NP_002511.1::p.W288Cfs*12	0.00071	Positive	Type A	NM_002520.6:c.860_863dup	NP_002511.1:p.Trp288CysfsTer12	0.00321	Positive	Not Detected	0
CH012372	Positive	Type A	NM_002520.7:c.860_863dup	NP_002511.1::p.W288Cfs*12	0.00971	Positive	Type A	NM_002520.6:c.860_863dup	NP_002511.1:p.Trp288CysfsTer12	0.01283	Positive	Positive	0.00571
CH012379	Positive	Type D	NM_002520.7:c.863_864insCCTG	NP_002511.1::p.W288Cfs*12	0.00426	Positive	Type D	NM_002520.6:c.863_864insCCTG	NP_002511.1:p.Trp288CysfsTer12	0.01686	Negative	Not Tested	NA
CH012419	Positive	Type A	NM_002520.7:c.860_863dup	NP_002511.1::p.W288Cfs*12	0.00066	Not Detected	NA	NA	NA	NA	Negative	Not Tested	NA
CH012427	Positive	Type A	NM_002520.7:c.860_863dup	NP_002511.1::p.W288Cfs*12	0.00194	Positive	Type A	NM_002520.6:c.860_863dup	NP_002511.1:p.Trp288CysfsTer12	0.00263	Positive	Positive	0.000716
CH012451	Positive	Type B	NM_002520.7:c.863_864insCATG	NP_002511.1::p.W288Cfs*12	0.02110	Positive	Type B	NM_002520.6:c.863_864insCATG	NP_002511.1:p.Trp288CysfsTer12	0.01058	Positive	Positive	0.0113
CH012452	Positive	Type A	NM_002520.7:c.860_863dup	NP_002511.1::p.W288Cfs*12	0.00126	Not Detected	NA	NA	NA	NA	Negative	Not Tested	NA
CH012493	Positive	Type A	NM_002520.7:c.860_863dup	NP_002511.1::p.W288Cfs*12	0.08600	Positive	Type A	NM_002520.6:c.860_863dup	NP_002511.1:p.Trp288CysfsTer12	0.10001	Positive	Positive	0.223
CH012495	Not Detected	NA	NA	NA	NA	Positive	Other	NM_002520.6:c.863_864insTCGG	NP_002511.1:p.Trp288CysfsTer12	0.00352	Negative	Not Tested	NA
CH012499	Positive	Type A	NM_002520.7:c.860_863dup	NP_002511.1::p.W288Cfs*12	0.00165	Not Detected	NA	NA	NA	NA	Positive	Positive	0.000589
CH012506	Positive	Type A	NM_002520.7:c.860_863dup	NP_002511.1::p.W288Cfs*12	0.00334	Not Detected	NA	NA	NA	NA	Negative	Not Tested	NA
CH012512	Positive	Type A	NM_002520.7:c.860_863dup	NP_002511.1::p.W288Cfs*12	0.00122	Not Detected	NA	NA	NA	NA	Positive	Not Detected	0
CH012519	Positive	Type A	NM_002520.7:c.860_863dup	NP_002511.1::p.W288Cfs*12	0.00112	Not Detected	NA	NA	NA	NA	Positive	Not Detected	0
CH012551	Positive	Type A	NM_002520.7:c.860_863dup	NP_002511.1::p.W288Cfs*12	0.04960	Positive	Type A	NM_002520.6:c.860_863dup	NP_002511.1:p.Trp288CysfsTer12	0.04948	Negative	Not Tested	NA
CH012555	Positive	Type A	NM_002520.7:c.860_863dup	NP_002511.1::p.W288Cfs*12	0.00035	Not Detected	NA	NA	NA	NA	Negative	Not Tested	NA
CH014344	Positive	Type A	NM_002520.7:c.860_863dup	NP_002511.1::p.W288Cfs*12	0.00128	Not Detected	NA	NA	NA	NA	Positive	Not Detected	0
CH016377	Positive	Type A	NM_002520.7:c.860_863dup	NP_002511.1::p.W288Cfs*12	0.00124	Not Detected	NA	NA	NA	NA	Positive	Not Detected	0
CH016397	Positive	Type A	NM_002520.7:c.860_863dup	NP_002511.1::p.W288Cfs*12	0.00046	Not Detected	NA	NA	NA	NA	Positive	Not Detected	0
CH016455	Not Detected	NA	NA	NA	NA	Not Detected	NA	NA	NA	NA	Positive	Positive	0.000552
CH016519	Positive	Type A	NM_002520.7:c.860_863dup	NP_002511.1::p.W288Cfs*12	0.02560	Positive	Type A	NM_002520.6:c.860_863dup	NP_002511.1:p.Trp288CysfsTer12	0.04923	Positive	Positive	0.0351
CH016524	Positive	Type A	NM_002520.7:c.860_863dup	NP_002511.1::p.W288Cfs*12	0.00065	Not Detected	NA	NA	NA	NA	Positive	Not Detected	0
CH016559	Positive	Type A	NM_002520.7:c.860_863dup	NP_002511.1::p.W288Cfs*12	0.00239	Not Detected	NA	NA	NA	NA	Positive	Positive	0.000373
CH016593	Positive	Type A	NM_002520.7:c.860_863dup	NP_002511.1::p.W288Cfs*12	0.03810	Positive	Type A	NM_002520.6:c.860_863dup	NP_002511.1:p.Trp288CysfsTer12	0.06740	Positive	Positive	0.00375
CH016620	Not Detected	NA	NA	NA	NA	Positive	Type A	NM_002520.6:c.860_863dup	NP_002511.1:p.Trp288CysfsTer12	0.00306	Positive	Not Detected	0
CH016653	Positive	Other	NM_002520.7:c.863_864insCCGG	NP_002511.1::p.W288Cfs*12	0.00351	Not Detected	NA	NA	NA	NA	Negative	Not Tested	NA
CH016678	Positive	Type D	NM_002520.7:c.863_864insCCTG	NP_002511.1::p.W288Cfs*12	1.50000	Positive	Type D	NM_002520.6:c.863_864insCCTG	NP_002511.1:p.Trp288CysfsTer12	1.36936	Negative	Not Tested	NA
CH016701	Positive	Type A	NM_002520.7:c.860_863dup	NP_002511.1::p.W288Cfs*12	0.04070	Positive	Type A	NM_002520.6:c.860_863dup	NP_002511.1:p.Trp288CysfsTer12	0.03321	Negative	Not Tested	NA
CH016717	Positive	Type D	NM_002520.7:c.863_864insCCTG	NP_002511.1::p.W288Cfs*12	0.00160	Not Detected	NA	NA	NA	NA	Positive	Positive	0.000858
CH016759	Not Detected	NA	NA	NA	NA	Positive	Type A	NM_002520.6:c.860_863dup	NP_002511.1:p.Trp288CysfsTer12	0.01243	Positive	Not Detected	0
CH016803	Positive	Type B	NM_002520.7:c.863_864insCATG	NP_002511.1::p.W288Cfs*12	0.00209	Not Detected	NA	NA	NA	NA	Positive	Not Detected	0
CH016817	Positive	Type A	NM_002520.7:c.860_863dup	NP_002511.1::p.W288Cfs*12	0.00024	Not Detected	NA	NA	NA	NA	Negative	Not Tested	NA
CH016830	Not Detected	NA	NA	NA	NA	Not Detected	NA	NA	NA	NA	Positive	Positive	0.00396
CH016837	Positive	Other	NM_002520.7:c.863_864insTCGG	NP_002511.1::p.W288Cfs*12	0.00520	Positive	Other	NM_002520.6:c.863_864insTCGG	NP_002511.1:p.Trp288CysfsTer12	0.00263	Negative	Not Tested	NA
CH016856	Positive	Type D	NM_002520.7:c.863_864insCCTG	NP_002511.1::p.W288Cfs*12	0.83700	Positive	Type D	NM_002520.6:c.863_864insCCTG	NP_002511.1:p.Trp288CysfsTer12	1.11028	Positive	Positive	1.62
CH016880	Positive	Type A	NM_002520.7:c.860_863dup	NP_002511.1::p.W288Cfs*12	0.00255	Not Detected	NA	NA	NA	NA	Positive	Positive	0.00119
CH016921	Positive	Type D	NM_002520.7:c.863_864insCCTG	NP_002511.1::p.W288Cfs*12	0.13500	Positive	Type D	NM_002520.6:c.863_864insCCTG	NP_002511.1:p.Trp288CysfsTer12	0.11194	Negative	Not Tested	NA
CH016923	Not Detected	NA	NA	NA	NA	Not Detected	NA	NA	NA	NA	Positive	Positive	0.000321
CH016930	Positive	Type A	NM_002520.7:c.860_863dup	NP_002511.1::p.W288Cfs*12	0.00140	Positive	Type A	NM_002520.6:c.860_863dup	NP_002511.1:p.Trp288CysfsTer12	0.00581	Positive	Positive	0.00347
CH016953	Positive	Type D	NM_002520.7:c.863_864insCCTG	NP_002511.1::p.W288Cfs*12	0.00082	Not Detected	NA	NA	NA	NA	Positive	Not Detected	0
CH016955	Positive	Type A	NM_002520.7:c.860_863dup	NP_002511.1::p.W288Cfs*12	0.00334	Not Detected	NA	NA	NA	NA	Positive	Not Detected	0
CH016981	Positive	Other	NM_002520.7:c.867_868insAGGT	NP_002511.1::p.W290Rfs*10	0.52400	Positive	Other	NM_002520.6:c.867_868insAGGT	NP_002511.1:p.Trp290ArgfsTer10	0.61296	Positive	Positive	0.995
CH016996	Positive	Type D	NM_002520.7:c.863_864insCCTG	NP_002511.1::p.W288Cfs*12	0.11200	Positive	Type D	NM_002520.6:c.863_864insCCTG	NP_002511.1:p.Trp288CysfsTer12	0.11455	Positive	Positive	0.148
CH017039	Positive	Type A	NM_002520.7:c.860_863dup	NP_002511.1::p.W288Cfs*12	0.00160	Not Detected	NA	NA	NA	NA	Positive	Positive	0.000856
CH017063	Positive	Type D	NM_002520.7:c.863_864insCCTG	NP_002511.1::p.W288Cfs*12	0.00189	Not Detected	NA	NA	NA	NA	Negative	Not Tested	NA
CH012150	Positive	Type D	NM_002520.7:c.863_864insCCTG	NP_002511.1::p.W288Cfs*12	0.00224	Not Detected	NA	NA	NA	NA	Negative	Not Tested	NA
CH012321	Positive	Type D	NM_002520.7:c.863_864insCCTG	NP_002511.1::p.W288Cfs*12	0.00063	Not Detected	NA	NA	NA	NA	Positive	Not Detected	0
CH012487	Positive	Type D	NM_002520.7:c.863_864insCCTG	NP_002511.1::p.W288Cfs*12	0.00247	Not Detected	NA	NA	NA	NA	Positive	Not Detected	0
CH012535	Not Detected	NA	NA	NA	NA	Positive	Type A	NM_002520.6:c.860_863dup	NP_002511.1:p.Trp288CysfsTer12	0.00417	Negative	Not Tested	NA
CH012186	Positive	Type A	NM_002520.7:c.860_863dup	NP_002511.1::p.W288Cfs*12	0.02350	Positive	Type A	NM_002520.6:c.860_863dup	NP_002511.1:p.Trp288CysfsTer12	0.00854	Negative	Not Tested	NA
CH012205	Positive	Type A	NM_002520.7:c.860_863dup	NP_002511.1::p.W288Cfs*12	0.00137	Not Detected	NA	NA	NA	NA	Positive	Positive	0.000707
CH012215	Positive	Type A	NM_002520.7:c.860_863dup	NP_002511.1::p.W288Cfs*12	1.37000	Positive	Type A	NM_002520.6:c.860_863dup	NP_002511.1:p.Trp288CysfsTer12	1.29507	Positive	Positive	0.569
CH012349	Positive	Other	NM_002520.7:c.863_864insCCAG	NP_002511.1::p.W288Cfs*12	0.00181	Not Detected	NA	NA	NA	NA	Positive	Not Detected	0
CH012505	Positive	Other	NM_002520.7:c.863_864insCTTG	NP_002511.1::p.W288Cfs*12	0.01330	Not Detected	NA	NA	NA	NA	Positive	Positive	0.0094
CH016601	Positive	Type D	NM_002520.7:c.863_864insCCTG	NP_002511.1::p.W288Cfs*12	0.00143	Not Detected	NA	NA	NA	NA	Positive	Positive	0.00208
CH016656	Not Detected	NA	NA	NA	NA	Not Detected	NA	NA	NA	NA	Positive	Positive	0.00133
CH016670	Positive	Type A	NM_002520.7:c.860_863dup	NP_002511.1::p.W288Cfs*12	0.00111	Not Detected	NA	NA	NA	NA	Positive	Positive	0.00225
CH016801	Positive	Type A	NM_002520.7:c.860_863dup	NP_002511.1::p.W288Cfs*12	2.10000	Positive	Type A	NM_002520.6:c.860_863dup	NP_002511.1:p.Trp288CysfsTer12	1.87443	Positive	Positive	0.421
CH016874	Positive	Type A	NM_002520.7:c.860_863dup	NP_002511.1::p.W288Cfs*12	0.00610	Positive	Type A	NM_002520.6:c.860_863dup	NP_002511.1:p.Trp288CysfsTer12	0.00872	Positive	Positive	0.0046
CH016935	Positive	Type A	NM_002520.7:c.860_863dup	NP_002511.1::p.W288Cfs*12	0.00245	Not Detected	NA	NA	NA	NA	Positive	Not Detected	0
CH016948	Not Detected	NA	NA	NA	NA	Positive	Type A	NM_002520.6:c.860_863dup	NP_002511.1:p.Trp288CysfsTer12	0.00814	Negative	Not Tested	NA
CH017021	Positive	Type A	NM_002520.7:c.860_863dup	NP_002511.1::p.W288Cfs*12	0.00909	Positive	Type A	NM_002520.6:c.860_863dup	NP_002511.1:p.Trp288CysfsTer12	0.00401	Positive	Positive	0.00422
CH017057	Not Detected	NA	NA	NA	NA	Positive	Type A	NM_002520.6:c.860_863dup	NP_002511.1:p.Trp288CysfsTer12	0.01125	Negative	Not Tested	NA
CH016785	Positive	Type A	NM_002520.7:c.860_863dup	NP_002511.1::p.W288Cfs*12	0.00099	Not Detected	NA	NA	NA	NA	Positive	Positive	0.0165
CH012367	Positive	Type A	NM_002520.7:c.860_863dup	NP_002511.1::p.W288Cfs*12	0.55700	Positive	Type A	NM_002520.6:c.860_863dup	NP_002511.1:p.Trp288CysfsTer12	0.52995	Negative	Not Tested	NA
CH012289	Positive	Type A	NM_002520.7:c.860_863dup	NP_002511.1::p.W288Cfs*12	0.30200	Positive	Type A	NM_002520.6:c.860_863dup	NP_002511.1:p.Trp288CysfsTer12	0.51069	Positive	Not Detected	0
CH016504	Positive	Type A	NM_002520.7:c.860_863dup	NP_002511.1::p.W288Cfs*12	0.00094	Not Detected	NA	NA	NA	NA	Positive	Positive	0.00156
CH016611	Positive	Type B	NM_002520.7:c.863_864insCATG	NP_002511.1::p.W288Cfs*12	0.00916	Not Detected	NA	NA	NA	NA	Negative	Not Tested	NA
CH016671	Positive	Type A	NM_002520.7:c.860_863dup	NP_002511.1::p.W288Cfs*12	0.01610	Positive	Type A	NM_002520.6:c.860_863dup	NP_002511.1:p.Trp288CysfsTer12	0.02584	Positive	Not Detected	0
CH016725	Positive	Type A	NM_002520.7:c.860_863dup	NP_002511.1::p.W288Cfs*12	0.24600	Positive	Type A	NM_002520.6:c.860_863dup	NP_002511.1:p.Trp288CysfsTer12	0.16481	Negative	Not Tested	NA
CH016826	Positive	Type B	NM_002520.7:c.863_864insCATG	NP_002511.1::p.W288Cfs*12	0.09330	Positive	Type B	NM_002520.6:c.863_864insCATG	NP_002511.1:p.Trp288CysfsTer12	0.08969	Negative	Not Tested	NA
CH016964	Positive	Type A	NM_002520.7:c.860_863dup	NP_002511.1::p.W288Cfs*12	0.00070	Positive	Type A	NM_002520.6:c.860_863dup	NP_002511.1:p.Trp288CysfsTer12	0.00905	Positive	Not Detected	0

IVS, Invivoscrive; MRD, measurable residual disease; VAF, variant allele fraction; AMP, anchored multiplex PCR, ITD, internal tandem duplication; NA, not applicable

References

1. Dillon LW, Gui G, Page KM, Ravindra N, Wong ZC, Andrew G*, et al.* DNA Sequencing to Detect Residual Disease in Adults With Acute Myeloid Leukemia Prior to Hematopoietic Cell Transplant. *JAMA* 2023 Mar 7; **329**(9)**:** 745-755.
